# Lumican and versican protein expression are associated with colorectal adenoma-to-carcinoma progression

**DOI:** 10.1371/journal.pone.0174768

**Published:** 2017-05-08

**Authors:** Meike de Wit, Beatriz Carvalho, Pien M. Delis-van Diemen, Carolien van Alphen, Jeroen A. M. Beliën, Gerrit A. Meijer, Remond J. A. Fijneman

**Affiliations:** 1 Department of Pathology, Netherlands Cancer Institute, Amsterdam, the Netherlands; 2 Department of Pathology, VU University Medical Center, Amsterdam, the Netherlands; 3 Department of Medical Oncology, VU University Medical Center, Amsterdam, the Netherlands; University of Crete, GREECE

## Abstract

**Background:**

One prominent event associated with colorectal adenoma-to-carcinoma progression is genomic instability. Approximately 85% of colorectal cancer cases exhibit chromosomal instability characterized by accumulation of chromosome copy number aberrations (CNAs). Adenomas with gain of chromosome 8q, 13q, and/or 20q are at high risk of progression to cancer. Tumor progression is also associated with expansion of the extracellular matrix (ECM) and the activation of non-malignant cells within the tumor stroma. The glycoproteins versican and lumican are overexpressed at the mRNA level in colon carcinomas compared to adenomas, and are associated with the formation of tumor stroma.

**Purpose:**

The aim of this study was to characterize versican and lumican protein expression in tumor progression and investigate their association with CNAs commonly associated with adenoma-to-carcinoma progression.

**Methods:**

Tissue microarrays were constructed with colon adenomas and carcinomas that were characterized for MSI-status and DNA copy number gains of chromosomes 8q, 13q and 20q. Sections were immunohistochemically stained for lumican and versican. Protein expression levels were evaluated using digitized slides, and scores were finally dichotomized into a positive or negative score per sample.

**Results:**

Lumican and versican expression were both observed in neoplastic cells and in the tumor stroma of colon adenomas and carcinomas. Lumican expression was more frequently present in epithelial cells of carcinomas than adenomas (49% versus 18%; *P* = 0.0001) and in high-risk adenomas and carcinomas combined compared to low-risk adenomas (43% versus 16%; *P* = 0.005). Versican staining in the tumor stroma was more often present in high-risk adenomas combined with carcinomas compared to low-risk adenomas (57% versus 36%; *P* = 0.03) and was associated with the presence of gain of 13q (71% versus 44%; *P* = 0.04).

**Conclusion:**

Epithelial lumican and stromal versican protein expression are increased during colorectal adenoma-to-carcinoma progression.

## Introduction

Colorectal cancer (CRC) develops in a multistep process that starts with the formation of a colon adenoma. These are benign lesions arising from the intestinal epithelium, of which only a small subset progresses into CRC [1,2]. The progression of normal colon epithelium to invasive CRC is accompanied with (epi)genetic changes that cause alterations in cellular processes resulting in tumorigenic capacities [3]. Specific gains and losses of (parts of) chromosomes are associated with adenoma-to-carcinoma progression [4], and these include gains of 20q, 13q and 8q [5]. These chromosomal aberrations are considered non-random events that drive adenoma-to carcinoma progression [6–8].

In addition to changes in neoplastic cells, the formation of a tumor-specific microenvironment contributes to tumor progression. One of the most prominent changes in colorectal adenoma-to-carcinoma progression is the expansion of the extracellular matrix (ECM) accompanied by activation of non-malignant cells within the tumor stroma. This is reflected by an altered histological presentation hallmarked by (myo)fibroblast proliferation, macrophage and lymphocyte infiltration, and outgrowth of blood vessels [9]. The interplay between cancer cells and this tumor stroma leads to production of growth signals as well as survival signals to evade apoptosis, facilitates migration and metastasis through remodeling of the ECM, and stimulates angiogenesis for a constant supply of oxygen and nutrients [10]. The extent to which the surrounding ECM influences the progression of colon adenomas to CRC and later development of CRC metastasis remains to be resolved, although several studies suggest an important role. For example, desmoplastic changes were correlated with disease recurrence in CRC stage II patients [11]. In addition, specific genomic alterations in CRC cells are associated with the stroma percentage and have prognostic value [12–14].

Previously we performed a genome-wide mRNA expression study, which revealed genes whose expression was upregulated in carcinomas compared to adenomas [15]. A subsequent pathway analysis of this dataset revealed that ‘stroma activation’ was one of the cancer related gene sets significantly upregulated in carcinomas compared to adenomas [16]. Among the genes that were upregulated in carcinomas, several well known stroma-associated glycoproteins were present, including lumican and versican. Lumican (gene symbol: *LUM*) is a member of the small leucine rich proteoglycan family and has a role in fibrillar network formation. Lumican is thought to modify the fibrous tissue surrounding a tumor, thereby inducing a tumor-specific ECM [17]. Versican (gene symbol: *VCAN*, also known as *CSPG2*) belongs to the family of large chondroitin sulfate proteoglycans and has hyaluronate binding properties [18]. Versican is thought to play a role in the formation of an anti-adhesive ECM that would support cancer cell growth and metastasis, and to stimulate production of pro-inflammatory cytokines by monocytes [19,20]. Previously, we showed that versican and lumican protein expression are associated to a longer survival of stage II and III colon cancer patients, respectively [21]. In the present study we investigated lumican and versican protein expression in adenoma-to-carcinoma progression and their potential association with commonly occurring genomic alterations involved in that process.

## Materials and methods

### Patients and tissue material

Tissue micro arrays (TMAs) were constructed previously [22] using a series of 82 colon adenomas and 82 carcinomas that were collected retrospectively from 2001 to 2008 at the VU University Medical Center, Amsterdam, The Netherlands. Collection, storage, and use of tissue and patient data were performed in accordance with the Code for proper secondary use of human tissue in the Netherlands [23,24]. Two to six cores from each paraffin block were included in the TMAs, depending on the amount of tissue available.

Tumor samples were previously characterized for microsatellite instability (MSI) status and DNA copy number aberrations (CNAs) of chromosomal regions 8q, 13q and 20q [22]. For associations between presence or absence of CNAs and protein expression, only microsatellite stable (MSS) lesions were considered. Adenomas that were MSS and showed a gain in one or more of these chromosomal regions were classified as high-risk adenomas while MSS adenomas without these gains were classified as low-risk adenomas.

### Lumican and versican immunohistochemistry

Tissue sections were deparaffinized and rehydrated and endogenous peroxidases were blocked with 0.3% hydrogen peroxide in methanol. For the versican staining, antigens were retrieved by microwaving for 30 minutes at 90 watt in 10mM citrate buffer solution (pH 6.0). Primary mouse anti-versican antibody (clone 2-B-1, Seikagaku, Tokyo, Japan) was incubated at a 1:300 dilution in PBS containing 1% BSA and 0.1% Tween-20 (Sigma-Aldrich, St Louis, USA) at 4°C overnight, and subsequently detected by a HRP-coupled anti-mouse polymer (Envision, DAKO, Heverlee, Belgium) and incubation with Diaminobenzidine (DAKO). For the lumican staining, antigen retrieval was done by autoclaving in Tris (10mM)/EDTA (1mM) buffer (pH 9.0). The primary rabbit anti-lumican antibody (HPA001522, atlas antibodies, Stockholm, Sweden) was incubated at a dilution of 1:50 in antibody dilutend (DAKO) overnight at 4°C. Staining was detected by incubation with a HRP-coupled anti-rabbit polymer (Envision, DAKO) and incubation with Diaminobenzidine (DAKO). All sections were counterstained with Mayer’s haematoxylin.

### Analysis of lumican and versican stainings

The stained TMA sections were automatically scanned with a digital pathology system (Mirax slide Scanner system 3DHISTECH Ltd.,Budapest, Hungary) equipped with a 20x objective with a numerical aperture of 0.75 (Carl Zeiss B.V., Sliedrecht, the Netherlands). Epithelial staining and stromal staining were scored separately into 4 categories; negative, weak, moderate or strong staining. For lumican, the highest score over multiple cores from each tumor was used for further analysis. For versican, because of its strong staining, the lowest score of multiple cores for each tumor was used for further analysis. Final evaluation was performed by dichotimizing the data into a negative and a positive group. Due to loss of cores for technical reasons during the staining procedure, results could be evaluated for fewer than the 82 carcinoma tissues and 82 adenoma tissues included. For lumican staining 72 colon adenomas and 65 colon carcinomas were evaluated, for versican 75 colon adenomas and 71 carcinomas (Tables 1 and 2).

**Table 1:** Distribution of lumican staining according to histological and molecular characteristics of the colorectal adenoma and carcinoma samples. MSS: microsatellite stable, MSI: microsatellite instable, CS: chromosomal stable, CIN: chromosomal instable, CNA: copy number aberration.

	Epithelial lumican staining			Stromal lumican staining		
	Negative	Positive	P-value	Negative	Positive	P-value
**Lesion n(%)**						
adenomas	59 (82)	13 (18)		20 (28)	52 (72)	
carcinomas	33 (51)	32 (49)	0.0001**	14 (22)	51 (78)	0.4*
**Histological type n(%)**						
Tubular	32 (80)	8 (20)		8 (20)	32 (80)	
Tubulovillous	25 (83)	5 (17)		12 (40)	18 (60)	
Villous	2 (100)	0 (0)	0.8**	0	2 (100)	0.2**
**Dysplasia n(%)**						
Low-grade	50 (81)	12 (19)		14 (23)	48 (77)	
High-grade	9 (90)	1 (10)	0.7**	6 (60)	4 (40)	0.06**
**Differentiation grade n(%)**						
Poor	4 (57)	3 (43)		1 (14)	6 (86)	
Moderate	28 (49)	29 (51)		13 (23)	44 (77)	
Well	1 (100)	0 (0)	1.0**	0	1 (100)	1.0**
**Dukes stage n(%)**						
A	19 (73)	7 (27)		8 (31)	18 (69)	
B	9 (37)	15 (63)		3 (13)	21 (87)	
C	5 (36)	9 (64)		3 (21)	11 (79)	
D	0 (0)	1 (100)	0.02**	0	1 (100)	0.5**
**Microsattelite instability n(%)**						
MSS	79 (68)	38 (32)		31 (26)	86 (74)	
MSI	6 (67)	3 (33)	1.0**	2 (22)	7 (78)	1.0**
**Chromosomal instable n(%)**						
adenomas (CS), MSS	7 (16)	36 (84)		11 (26)	32 (74)	
adenomas (CIN) + carcinomas, MSS	25 (43)	33 (57)	0.005*	12 (21)	46 (79)	0.6*
**Chromosomal changes all MSS lesions n(%)**						
Gain of 8q	7 (41)	10 (59)		4 (24)	13 (76)	
No gain of 8q	62 (74)	22 (26)	0.01*	18 (22)	65 (78)	1.0**
Gain of 13q	15 (54)	13 (46)		3 (11)	25 (89)	
No gain of 13q	54 (74)	19 (26)	0.06*	20 (27)	53 (73)	0.1*
Gain of 20q	18 (50)	18 (50)		5 (14)	31 (86)	
No gain of 20q	51 (78)	14 (22)	0.004*	18 (28)	47 (72)	0.1*
**Chromosomal changes CNA lesions n(%)**						
Gain of 8q	7 (41)	10 (59)		4 (24)	13 (76)	
No gain of 8q	26 (63)	15 (37)	0.2*	8 (20)	33 (80)	1.0**
Gain of 13q	15 (54)	13 (46)		3 (11)	25 (89)	
No gain of 13q	18 (60)	12 (40)	0.8*	9 (30)	21 (70)	0.1*
Gain of 20q	18 (50)	18 (50)		5 (14)	31 (86)	
No gain of 20q	15 (68)	7 (32)	0.3*	7 (32)	15 (68)	0.2**
**Gender n(%)**						
Male	52 (69)	23 (31)		19 (25)	56 (75)	
Female	4 (57)	3 (43)	0.6*	20 (28)	52 (72)	1.0*
**Age (mean(sd))**	71.8 (9.9)	65.7 (11.3)	**0.002^**	70.6 (9.7)	69.5 (11.0)	0.5**^**

*Pearson Chi-Square,2-sided exact

**Fishers's exact test

^student t-test

**Table 2:** Distribution of versican staining according to histological and molecular characteristics of the colorectal adenoma and carcinoma samples. MSS: microsatellite stable, MSI: microsatellite instable, CS: chromosomal stable, CIN: chromosomal instable, CNA: copy number aberration.

	Epithelial versican staining			Stromal versican staining		
	Negative	Positive	P-value	Negative	Positive	P-value
**Lesion n(%)**						
adenomas	56 (75)	19 (25)		45 (60)	30 (40)	
carcinomas	56 (79)	15 (21)	0.6*	32 (45)	39 (55)	0.1*
**Histological type n(%)**						
Tubular	31 (76)	10 (24)		24 (59)	17 (41)	
Tubulovillous	22 (71)	9 (29)		20 (65)	11 (35)	
Villous	3 (100)	0 (0)	0.7**	1 (33)	2 (67)	0.6**
**Dysplasia n(%)**						
Low-grade	48 (75)	16 (25)		37 (58)	27 (42)	
High-grade	8 (73)	3 (27)	1.0**	8 (73)	3 (28)	0.5**
**Differentiation grade n(%)**						
Poor	8 (89)	1 (11)		3 (33)	6 (67)	
Moderate	48 (79)	13 (21)		29 (48)	32 (52)	
Well	0 (0)	1 (100)	0.2**	0	1 (100)	0.6**
**Dukes stage n(%)**						
A	23 (92)	3 (8)		15 (58)	11 (42)	
B	20 (71)	8 (29)		11 (39)	17 (61)	
C	13 (81)	3 (19)		6 (38)	10 (62)	
D	0 (0)	1 (100)	0.1**	0	1 (100)	0.3**
**Microsattelite Instability n(%)**						
MSS	97 (78)	27 (22)		66 (53)	58 (47)	
MSI	9 (82)	2 (18)	1.0**	7 (64)	4 (36)	0.6*
**Chromosomal instable n(%)**						
adenomas (CS), MSS	36 (80)	9 (20)		29 (64)	16 (36)	
adenomas (CIN) + carcinomas, MSS	47 (75)	16 (25)	0.7*	27 (43)	36 (57)	0.03*
**Chromosomal changes all MSS lesions n(%)**						
Gain of 8q	17 (89)	2 (11)		10 (53)	9 (47)	
No gain of 8q	66 (74)	23 (26)	0.2**	46 (52)	43 (48)	1.0*
Gain of 13q	21 (68)	10 (32)		9 (29)	22 (71)	
No gain of 13q	62 (81)	15 (19)	0.2*	47 (61)	30 (39)	0.003*
Gain of 20q	29 (71)	12 (29)		16 (39)	25 (61)	
No gain of 20q	54 (81)	13 (19)	0.3*	40 (60)	27 (40)	0.05*
**Chromosomal changes CNA lesions n(%)**						
Gain of 8q	17 (89)	2 (11)		10 (53)	9 (47)	
No gain of 8q	30 (68)	14 (32)	0.12**	17 (39)	27 (61)	0.41*
Gain of 13q	21 (68)	10 (32)		9 (29)	22 (71)	
No gain of 13q	26 (81)	6 (19)	0.2*	18 (56)	14 (44)	0.04*
Gain of 20q	29 (71)	12 (29)		16 (41)	25 (59)	
No gain of 20q	18 (82)	4 (18)	0.4*	11 (22)	11 (88)	0.4*
**Gender n(%)**						
Male	56 (72)	21 (28)		39 (51)	38 (49)	
Female	56 (81)	13 (19)	0.3*	38 (55)	31 (45)	0.6*
**Age (mean(sd))**	70,2 (10,6)	67,5 (12,8)	0.2^	71,1 (10,4)	67,8 (11,8)	0.07^

*Pearson Chi-Square,2-sided exact

**Fishers's exact test

^student t-test

### Statistical analysis

Pearson’s Chi-square test, or Fisher’s exact test whenever appropriate, was applied to evaluate associations between categorical variables; t-testing was applied for investigation of associations between the staining categories and age of the patients. All statistical tests were two sided, and *P*-values of < 0.05 were considered significant. All statistical analysis was performed by SPSS Statistics software, version 20 (SPSS, Chicago, IL, USA).

## Results

### Lumican and versican expression

**Fig 1:**
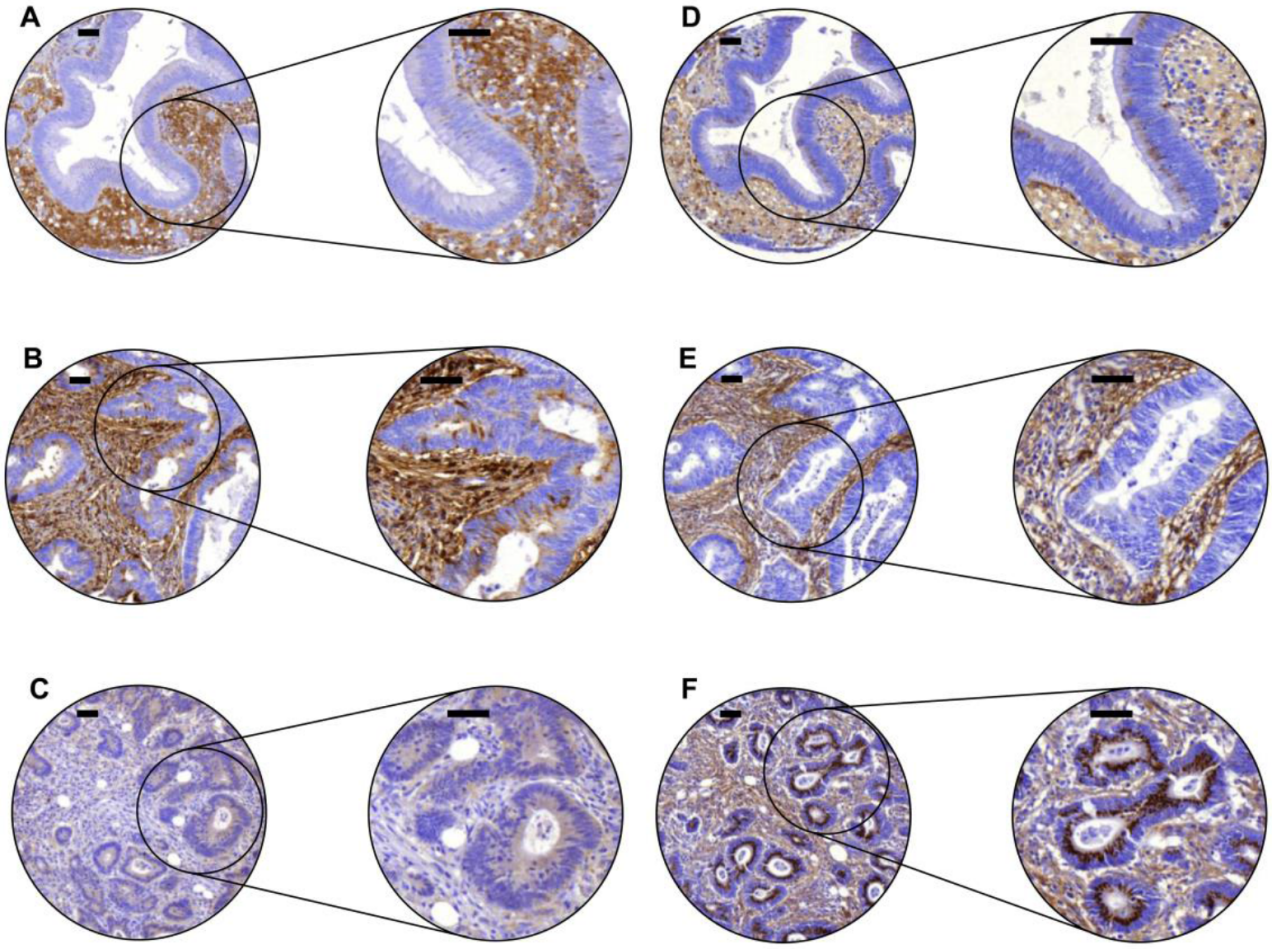
Lumican and versican expression in colon adenomas and carcinomas analyzed by immunohistochemistry. Representative examples show lumican staining (A-C) and versican staining (D-F) in the stroma and epithelium of the same tissue cores of one adenoma (A,D) and two carcinomas (B,C,E,F). These examples were classified as negative (0), weak (1), moderate (2), and strong (3), with expression for epithelium (E) and stroma (S) indicated between brackets [E,S] as follows; A [0.2], B [3.2], C [1.1], D [2.2], E [0.2], F [3.1]. Scale bars indicate 50 μm.

Protein expression of lumican and versican was observed in neoplastic epithelial cells as well as in the tumor stroma of both adenomas and carcinomas, representative examples are shown in Fig 1. Lumican epithelial staining was mostly cytoplasmic, often in combination with staining of the apical membrane. Lumican staining in the tumor stroma usually appeared as diffuse ECM staining, not confined to cells. Versican epithelial staining was also mostly cytoplasmic, sometimes with a granular staining pattern. In the tumor stroma endothelial- and (myo)fibroblast-like cells displayed versican positivity.

**Fig 2:**
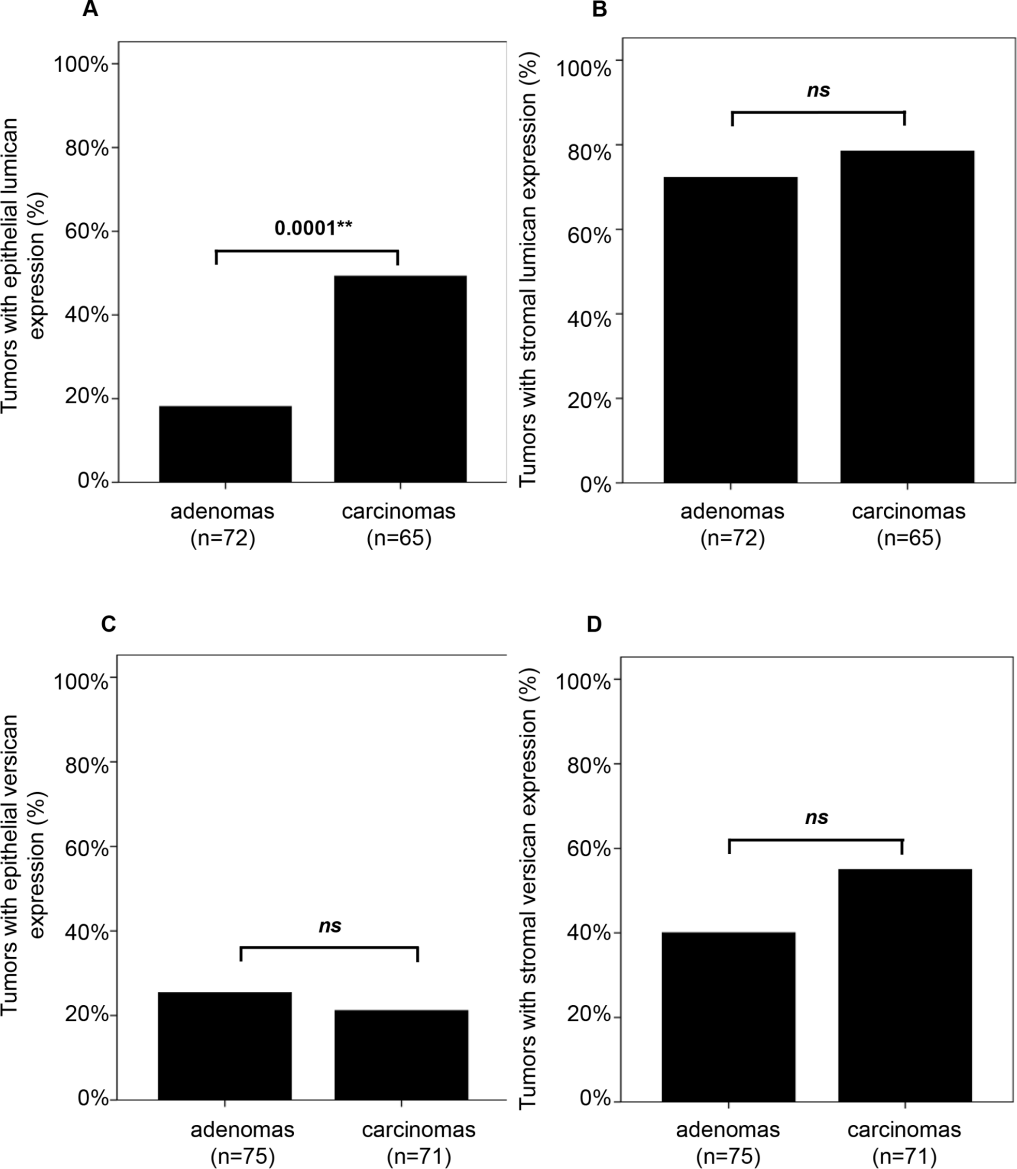
Lumican and versican expression compared between colon adenomas and carcinomas. Epithelial lumican staining was more frequently detected in carcinomas compared to adenomas (A). Stromal lumican staining was as frequent in colon adenomas as in colon carcinomas (B). The frequency of epithelial versican staining (C) and stromal versican staining (D) was similar in colon adenomas and carcinomas.

All epithelial and stromal scores for lumican and versican were dichotomized into a positive (weak, moderate and strong combined) and a negative group. Overall, epithelial lumican staining was present in 33% of all lesions and stromal expression was observed in 75% of cases (Table 1 and S1 Table). Lumican expression in the epithelium was significantly more often observed in carcinomas than in adenomas (49% *versus* 18%; *P* = 0.0001, Table 1 and Fig 2A and 2B), while stromal lumican expression was not associated with tumor progression. Epithelial versican staining was observed in 23% of all cases and stromal versican staining in 47% of cases (Table 2 and S1 Table). Neither versican expression in the epithelium nor in the stroma was significantly different between adenomas and carcinomas (Fig 2C and 2D).

Next, lumican and versican protein expression levels were compared to their mRNA expression levels in corresponding tumor samples. Previous work revealed lumican and versican as significantly higher expressed at the mRNA level by carcinomas compared to adenomas (S1A and S1B Fig, data from Carvalho *et al*., [7,15]). For approximately half of all tumor samples (50 and 53 for lumican and versican, respectively) such mRNA data were available. No significant correlations between mRNA and protein expression levels were found for lumican or versican in either the epithelial cells or tumor stroma or a combination of both.

### Lumican and versican expression in high-risk adenomas

**Fig 3:**
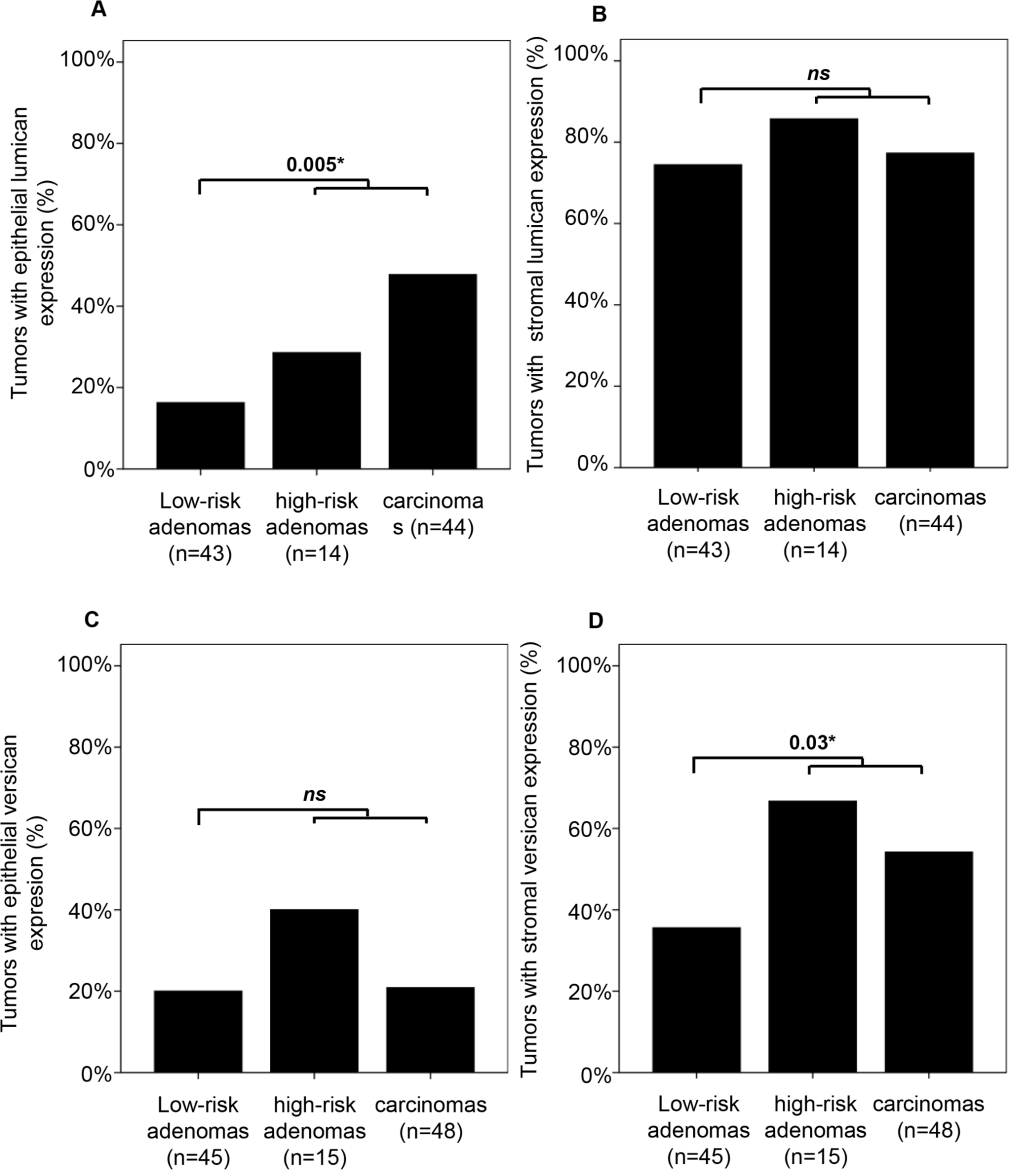
Lumican and versican expression in MSS low-risk adenomas, high-risk adenomas and carcinomas. Epithelial lumican staining was more frequently detected in high-risk adenomas combined with carcinomas compared to low-risk adenomas (A). The frequency of stromal lumican staining was similar for each type of lesion (B). The frequency of epithelial versican staining did also not differ between lesion types (C) while stromal versican staining was more frequently observed in high-risk adenomas combined with carcinomas compared to low-risk adenomas (D).

Chromosome 20q, 13q and 8q CNAs and MSI status had previously been determined for the majority of tumors in this cohort [22]. MSS (i.e. MSI negative) adenomas with a gain of chromosome 20q, 13q and/or 8q are at high risk of progressing to cancer and here defined as 'high-risk adenomas' compared to 'low-risk adenomas' that lack these CNAs. Lumican expression in neoplastic cells or in tumor stroma did not differ between high-risk adenomas compared to low-risk adenomas. When the group of high-risk adenomas was combined with carcinomas, epithelial lumican expression was more frequently observed compared to the group of low-risk adenomas (43% *versus* 16%; *P* = 0.005, Fig 3A). There was a trend for versican expression in tumor stroma to be more prevalent in high-risk adenomas compared to low-risk adenomas (67% *versus* 36%; *P* = 0.07, data not shown). When high-risk adenomas were combined with carcinomas, stromal versican staining was more frequently observed than in low-risk adenomas (57% *versus* 36%; *P* = 0.03, Fig 3D).

### Lumican and versican expression and gain of chromosome 8q, 13q, 20q

Next, we investigated whether lumican or versican expression was associated with one of the tumor-progression chromosome CNAs, i.e. gain of chromosome 8q, 13q, or 20q. For lumican this analysis was performed on 14 high-risk adenomas and 44 MSS carcinomas. None of the 8q, 13q, or 20q chromosomal aberrations was associated with lumican staining in the epithelial cells or tumor stroma (Table 1). For versican staining the same analyses were performed, with results for 15 high-risk adenomas and 48 MSS carcinomas. This revealed more versican staining in the stroma of tumors with gain of chromosome 13q compared to tumors without such a gain (71% *versus* 44%; *P* = 0.04, Table 2). Expression of lumican and versican was not associated with MSI status (Tables 1 and 2).

## Discussion

Lumican and versican are proteins relevant to the biology of cancer and their expression was found to be associated with clinical outcome. The present study aimed to explore the role of these proteins in the pathogenesis of CRC, and more explicitly their association with adenoma-to-carcinoma progression. To this end, expression levels of lumican and versican were explored in a series of 82 colorectal adenomas and 82 carcinomas. The expression of lumican was more prevalent in epithelial cells of CRCs than of adenomas. Versican staining was more often observed in the stroma of high-risk adenomas and carcinomas combined compared to low-risk adenomas. Although previous work revealed higher mRNA expression levels of the *LUM* and *VCAN* genes in carcinomas compared to adenomas, we did not observe significant correlations between mRNA and protein levels. Various post-transcriptional regulation mechanisms can lead to lack of correlation between mRNA expression levels and protein levels [25]. Alternatively, the absence of mRNA-protein correlation might also be explained by technical limitations of immunohistochemical stainings, which is a semi-quantitative rather than a quantitative read out of biology. Moreover, it is unknown what proportion of secreted lumican and versican is locally captured by the (tumor) tissue and still can be visualized by immunohistochemistry.

Accumulation of chromosomal aberrations is a potentially driving process in colorectal carcinogenesis and associated with high risk of adenoma-to-carcinoma progression; these chromosomal aberrations result in up- or down-regulation of many genes, via direct and indirect processes. The chromosomal regions of the *LUM* and *VCAN* genes (12q21.3-q22 and 5q14.3, respectively) are not among the most commonly gained regions in adenoma-to-carcinoma progression [5]. Lumican was upregulated in high-risk adenomas and CRCs, but its expression did not correlate to gain of any of the three chromosomal regions (8q, 13q, 20q) investigated in the present study. Lumican (as well as versican) expression was not correlated to MSI status either. We did find indications, however, that stromal expression of versican in high-risk adenomas and CRCs was associated with gain of chromosome 13q. This leads to the conclusion that lumican expression may be increased in a more progressed disease state independent of specific tumor progression-associated chromosomal aberrations, while increased stromal versican expression may be induced by factors directly regulated by gain of chromosome 13q.

A critical step in adenoma-to-carcinoma progression is invasion of tumor cells in the surrounding stroma while remodeling it into 'tumor stroma'. Lumican might have a dual role in cancer cells and stromal cells with opposite effects on cancer progression. For example, cell migration can be inhibited by lumican through prevention of the activation of focal adhesion kinase [26]. In addition, genes potentially regulating lumican include *HMGA2*, an oncogene that is highly expressed in about 50% of carcinomas [27], which can downregulate LUM expression by directly binding its promotor region [28]. These results led to the hypothesis that lumican may function as a tumor suppressor in the tumor stroma by inhibition of tumor cell motility and tumor plasticity [28]. However, also tumor-promoting effects have been reported. Lumican expression can result in less cell adhesion and loss of inhibition of cell proliferation via prevention of activation of the TGFB2/Smad2 pathway [29]. Furthermore, lumican secreted into cell culture medium may facilitate colon cancer cell migration [30]. A previous study by our group revealed that the presence of lumican in cancer cells was associated with a longer disease-free survival for stage II CRC patients [31]. This may indicate that the potential tumor suppressor role of lumican (in the tumor stroma) is less relevant in colon adenomas, and that expression of lumican is increased in adenoma-to-carcinoma progression in response to the development of tumor stroma. In line with this, the proportion of adenomas without lumican expression in the epithelium did not differ between the low-risk adenomas and the high-risk adenomas (84% and 71%, respectively).

In the current study, versican was increased in the tumor stroma of high-risk adenomas and carcinomas compared to low-risk adenomas, suggesting that versican plays a role in remodeling of tumor stroma in adenoma-to-carcinoma progression. Versican has been found to promote tumor development and metastasis through stimulation of cell proliferation and by increasing cell motility in several cancer types [32,33]. In contrast, versican is also capable to stimulate monocytes towards an anti-tumorigenic phenotype. Interestingly, this example of tumor-stroma interaction is more likely to happen for CRC than for breast cancer due to differences in versican expression by neoplastic cells from colon or breast origin [20]. Versican expression and secretion can also be controlled by secretion of TGFB and PDGF by epithelial tumor cells [34]. Considering the association of stromal versican expression with chromosome 13q gain, one or more factors on the 13q locus may (in)directly induce versican expression. For instance, chromosome 13q contains the miR-17-92 cluster and overexpression of this microRNA cluster is known to stimulate angiogenesis [35]. However, whether this or any other chromosome 13q-located factor can induce stromal versican expression remains to be determined.

In conclusion, in the present study protein expression of both lumican and versican was upregulated in high-risk adenomas and carcinomas compared to low-risk adenomas, indicating that they may play a role in adenoma-to-carcinoma progression. Further studies are required to elucidate the specific roles of these proteins and the mechanisms by which they influence adenoma-to-carcinoma progression.

## Supporting information

S1 Fig Expression microarray data of lumican and versican in colon adenomas and carcinomas. Box plots with dot plots of mRNA expression (determined by oligonucleotide microarrays) in colorectal adenomas and carcinomas [7,15]. For both lumican (A) and versican (B) mRNA expression was higher in carcinomas compared to adenomas.
(TIF)

S1 Table Participant-level data of versican and lumican staining, histological and molecular characteristics of the colorectal adenoma and carcinoma samples. MSS: microsatellite stable, MSI: microsatellite instable, gain: chromosomal gain.
(XLSX)
