# Supplementary figures and images for: Lumican and versican protein expression are associated with colorectal adenoma-to-carcinoma progression
Source: PLoS One. 2017 May 8;12(5):e0174768. doi: 10.1371/journal.pone.0174768 (PMC5421768; doi:10.1371/journal.pone.0174768)

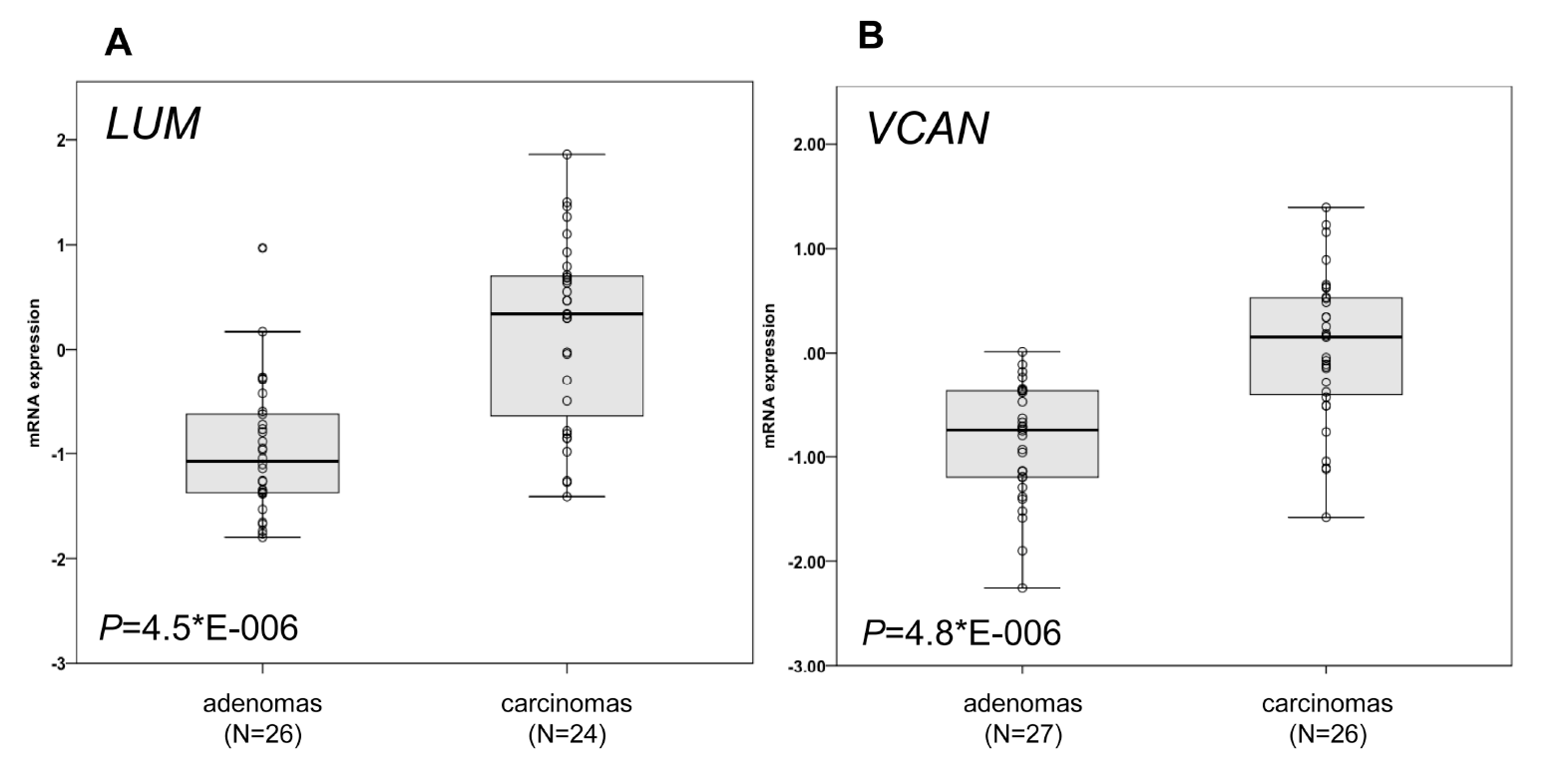

Supplement: S1 Fig — Box plots with dot plots of mRNA expression (determined by oligonucleotide microarrays) in colorectal adenomas and carcinomas [7,15]. For both lumican (A) and versican (B) mRNA expression was higher in carcinomas compared to adenomas. (TIF) [file pone.0174768.s001.tif]
